# Evidence for Increased High Alpha Intermuscular Coherence as a Measure of Reticulospinal Motor Drive

**DOI:** 10.1111/ejn.70517

**Published:** 2026-04-23

**Authors:** Nicole Sarah Holliger, Freschta Zipser‐Mohammadzada, Daniel Fabio Carpanese, Martin Schubert, Linard Filli

**Affiliations:** ^1^ Spinal Cord Injury Center Balgrist University Hospital, University of Zurich Zurich Switzerland; ^2^ Department of Health Sciences and Technology ETH Zurich Zurich Switzerland; ^3^ Neuroscience Center Zurich University of Zurich Zurich Switzerland; ^4^ Swiss Paraplegic Research Nottwil Switzerland

**Keywords:** EMG–EMG coherence, intermuscular coherence, postural control, reticulospinal, startle reflex, StartReact

## Abstract

The reticulospinal (RS) system is a key descending motor pathway involved in human movement control. However, it remains poorly characterized, largely due to methodological challenges in assessing its anatomy and physiology. Current approaches to gauge RS drive rely on intense stimuli (auditory cues, transcranial magnetic stimulation), limiting their feasibility. Here, we examined intermuscular coherence as a potential biomarker of common neural drive related to the RS system. Intermuscular coherence was evaluated during the startle reflex and StartReact paradigm, both known to be primarily mediated by the RS system. In 10 healthy participants, intermuscular coherence was analyzed across the sternocleidomastoid, lateral deltoid, and biceps brachii muscles bilaterally during the startle reflex. In 16 participants, intermuscular coherence was examined in the tibialis anterior (TA) during StartReact, as well as during postural perturbations, a postural balance task, and non‐postural ankle movements. Intermuscular coherence was enhanced across all muscles in the high alpha band during both the startle reflex and StartReact. A similar, albeit smaller peak in coherence was found at the same frequency during postural perturbations. TA intermuscular coherence was significantly enhanced at the high alpha and low beta bands during balance control vs. non‐postural ankle movements. The findings from the startle reflex and StartReact suggest that intermuscular coherence in the high alpha band reflects RS drive. RS contributions appear to be critical for postural control, particularly during balance‐related tasks, but not for non‐postural tasks involving TA. Results suggest that intermuscular coherence is a promising approach for assessing RS motor drive during movements.

## Graphical abstract

EMG–EMG coherence analyses were performed during acoustic startle reflex, StartReact, and dynamic movements of the lower limbs. Findings from the startle reflex and StartReact paradigm indicate that increased coherence in the high alpha band reflects reticulospinal drive. Our results suggest that the reticulospinal system plays a critical role in postural control, especially during balance‐related tasks and that intermuscular coherence analysis is a promising tool to quantify reticulospinal drive during movements.

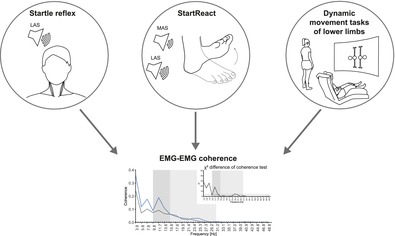

**Abbreviations**
BB: biceps brachii
CS: corticospinal
DL: lateral deltoid
EMG: electromyography
GM: gastrocnemius medialis
LAS: loud acoustic stimuli
MAS: moderate acoustic stimuli
RS: reticulospinal
SCM: sternocleidomastoid
TA: tibialis anterior
TAd: distal tibialis anterior
TAp: proximal tibialis anterior
TMS: transcranial magnetic stimulation

## 1 Introduction

The reticulospinal (RS) and corticospinal (CS) systems are the main descending motor tracts involved in human movement control. The CS system developed during the evolution of mammals and enables the execution of skilled, fine‐tuned movements of the hands and fingers (Lemon 2008; Lemon 2019). Most research on descending motor control has focused on the CS system, which may partially be attributed to the well‐known anatomy and various established methodologies for assessing (e.g., electroencephalography) or modulating (e.g., transcranial magnetic stimulation (TMS)) cortical activity in humans.

The RS system is a phylogenetically conserved motor pathway that contributes to elementary movements such as locomotion and posture (Schepens et al. 2008; Drew et al. 2004), as well as to motor control of arm and hand function (Baker and Perez 2017), including the coordination of bilateral hand movements (Dietz et al. 2024). Recent publications highlight the key role of the RS system in driving motor recovery after central nervous system damage such as spinal cord injury or stroke (Mooney et al. 2024; Sangari and Perez 2020). Compared with the CS system, the motor physiology of brainstem centers remains poorly understood, likely due to its diffuse anatomy and difficulty in accessing the RS system noninvasively. One approach that has recently gained attention for gauging RS drive involves the analysis of ipsilateral motor‐evoked potentials elicited by TMS over the primary motor cortex. There is compelling evidence that ipsilateral motor‐evoked potentials reflect cortico‐RS drive (Mooney et al. 2023; Ziemann et al. 1999; Taga et al. 2021). The principal approaches to assess RS drive rely on the application of loud acoustic stimuli (LAS): LAS trigger the acoustic startle reflex, a fast involuntary motor response primarily triggered by RS neurons in the caudal pons, resulting in a stereotypic activation of axial and proximal muscles (Koch 1999; Yeomans and Frankland 1996; Brown et al. 1991). LAS are also employed to investigate RS drive in the framework of the StartReact paradigm, which is characterized by a shortened reaction time when movement initiation is paired with a LAS (Valls‐Solé et al. 1995). Substantial evidence indicates that the StartReact effect is primarily mediated by the RS system triggering the fast release of preplanned motor programs (Valls‐Solé et al. 1999; Carlsen et al. 2004a; Neumann et al. 2025; Tapia et al. 2022). However, the mentioned approaches to gauge RS drive are dependent on stimulus‐evoked responses that may constrain their applicability during dynamic movements.

An alternative method circumventing these constraints is electromyographic (EMG) coherence analysis, which assesses synchronous oscillatory activity between two EMG signals, thereby providing evidence for common neural drive to the corresponding muscles (Farina et al. 2025). EMG–EMG coherence can be measured between EMG signals within the same muscle (intramuscular) or different muscles (intermuscular coherence). This approach has been widely used to gauge CS motor control. Enhanced muscular coherence in the low gamma band (35–60 Hz) has been reported during challenging motor tasks that strongly rely on cortical control (Clark et al. 2013; Jensen et al. 2018). In line with this finding, studies in stroke patients demonstrated a significant reduction in EMG–EMG coherence in the 35–40 Hz frequency (Lodha et al. 2017; Nielsen et al. 2008; Charalambous et al. 2024). During upper extremity tasks and tonic muscle contraction, CS drive has been associated with increased beta band (15–30 Hz) coherence (Kilner et al. 1999).

In contrast, the ability of EMG–EMG coherence to assess RS drive remains insufficiently understood. To date, only a limited number of studies have investigated EMG–EMG coherence as a potential readout of RS activity. One study reported enhanced intermuscular coherence between 10 and 20 Hz during acoustic startle responses, suggesting that this frequency range may reflect RS drive (Grosse and Brown 2003). Other studies have associated increased alpha band coherence (5–15 Hz) with RS activity during postural balance tasks or during movements of the impaired limb in stroke patients (Obata et al. 2014; Boonstra et al. 2009; Chen et al. 2018). A better understanding of the validity of EMG–EMG coherence as a measure of RS control is essential to advance its application for probing RS drive during dynamic movements without the need for external stimuli.

In this study, we aimed to characterize EMG–EMG coherence patterns across various muscles during two motor responses thought to be mediated by the RS system. First, we examined intermuscular coherence during the acoustic startle reflex. Second, EMG–EMG coherence was examined during the StartReact paradigm. An additional aim of this study was to investigate EMG–EMG coherence across various dynamic movement tasks of daily activity, including postural perturbation, balance control, and non‐postural ankle movements. We hypothesized that intermuscular coherence would be enhanced in the high alpha and low beta band during the two brainstem‐mediated motor responses (startle and StartReact), reflecting enhanced RS drive. Furthermore, we expected that RS drive would be enhanced during dynamic tasks requiring postural balance control. Alternative methodological approaches (e.g., EMG–EMG coherence) to quantitatively assess RS drive noninvasively and without intense, external stimuli (TMS, LAS) are essential to better understand the role of this fundamental motor system in movement control and during functional recovery after stroke or spinal cord injury.

## 2 Methods

### 2.1 Participants

Twenty‐six healthy participants participated in this study. Ten participants (6 female, age (mean ± standard deviation): 26.7 ± 1.9 years) participated in the first part of the study (Startle reflex) and sixteen participants (9 female, age: 26.5 ± 3.0 years) participated in the second part (StartReact, lower limb movement tasks). This study was approved by the Ethics Committee of the Canton Zurich (Study‐ID: 2021–00973) and is registered online (clinicaltrials.gov; NCT04967274). All experiments were conducted according to the guidelines of the Declaration of Helsinki and Good Clinical Practice. Written informed consent was obtained from all participants.

### 2.2 Experimental Setup and Procedures

#### 2.2.1 Startle Reflex

**FIGURE 1:**
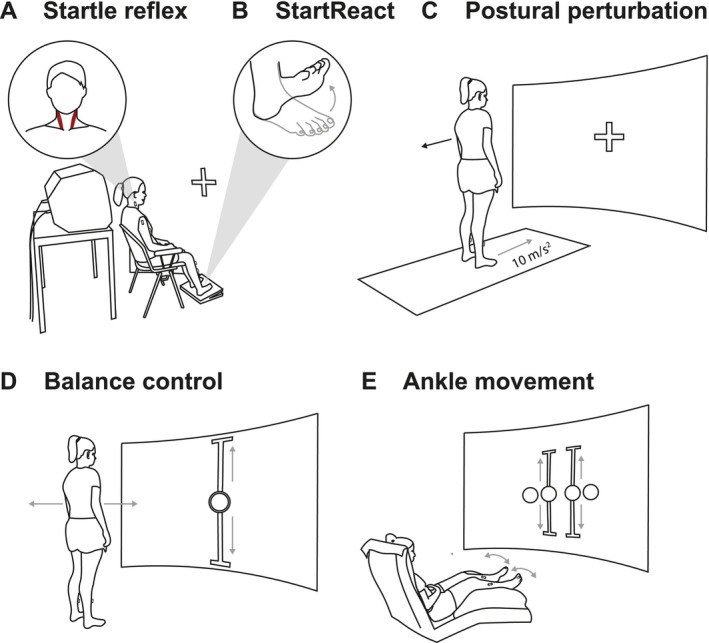
Schematic drawing of experimental procedures. (A) Experimental setup for startle reflex experiment. Participants sat on chair visually fixing a cross while LAS were applied by a loudspeaker. (B) Experimental setup for StartReact experiment. Participants were instructed to perform rapid, bilateral ankle dorsiflexions in response to MAS or LAS. (C) Experimental setup for postural perturbation task. Participants stood on a treadmill visually fixing a cross displayed in front of them. High‐acceleration perturbations were applied to simulate postural slips. (D) Experimental setup for balance control task. Participants were instructed to adapt their center of mass in anterior–posterior direction according to a projected, moving target. (E) Experimental setup for bilateral ankle movement task. Participants sat on a recliner and followed two synchronously moving visual targets with their feet.

Participants were seated comfortably in a chair while unexpected loud acoustic stimuli (LAS; 120 dB, 50 ms, 1000 Hz) were delivered through a loudspeaker (Electro‐Voice, ELX200, Burnsville, USA) positioned 0.3 m behind their heads. They were instructed to fixate a cross displayed in front of them and rest their hands on their thighs (Figure 1A). LAS were generated using digital‐to‐analog hardware (PCIe‐6321; National Instruments Inc., Austin, USA) controlled by a custom‐made Simulink script (Matlab R2021b, Mathworks Inc., Natick, USA). Before each experiment, sound intensity was calibrated and adjusted to 120 dB with a high‐precision sound level meter (CR162B, Cirrus Research plc; Hunmanby, UK). Initially, a single LAS was applied to familiarize participants with the stimulation. Subsequently, LAS were applied in varying, pseudo‐randomized intervals from 15 to 30 s to minimize stimulus predictability and facilitate startle responses. Four assessment blocks consisting of 10 LAS each were conducted, with five‐minute breaks between blocks to prevent muscle fatigue and maintain attention. Muscle activity was recorded bilaterally from the sternocleidomastoid (SCM), lateral deltoid (DL), and biceps brachii (BB). In a control task, participants were instructed to perform voluntary contractions of these muscles. For the SCM, participants were instructed to maintain a stable head position against a 1.5 kg load applied in a posterior direction via a head harness (GS trainer, Gatherer Systems, Suffolk, UK). Six assessment blocks were applied, with each block consisting of six contractions. Five‐minute breaks were taken between the blocks to prevent muscle fatigue. For the BB and DL muscles, participants performed six dynamic elbow flexion and shoulder abduction movements (Table 1).

**TABLE 1:** Overview of experimental protocol.

	Startle reflex	Voluntary contraction (control startle reflex)	StartReact	Postural perturbation (slips)	Ankle movement	Balance control
# of participants	10	10	16	16	16	16
# of experimental blocks	4	SCM: 6, DL: 1, BB: 1	4	2	2	2
Duration of each experimental block	4 min	SCM: 4 min, DL and BB: ~ 1 min	4 min	2 min	2.33 min	2.33 min
# Stimuli/Movements (per experimental block)	10 LAS (randomly applied)	SCM: 6 movements against load resistance DL: 6 dynamic shoulder abductions BB: 6 dynamic elbow flexions	10 LAS, 20 MAS (randomly applied)	6 treadmill belt accelerations in posterior direction (10 m/s^2^, duration: 300 ms, target speed: 1 m/s)	35 movements (0.25 Hz)	35 movements (0.25 Hz)
Target muscle (left and right side)	Sternocleidomastoid (SCM), Deltoideus—lateral part (DL), Biceps brachii (BB)	SCM, DL, BB	Tibialis anterior proximal (TAp), Tibialis anterior distal (TAd)	TAp	TAp	TAp
# segments analyzed in pooled coherence analysis	SCM: 141 DL: 29 BB: 24	SCM: 346 DL: 60 BB: 59	Intermuscular coherence LAS: 467 Intermuscular coherence MAS: 879 Intramuscular coherence LAS: 474 Intramuscular coherence MAS: 921	192	1084	764

#### 2.2.2 StartReact and Lower Limb Movement Tasks

For the StartReact experiments, participants were seated comfortably in a chair and instructed to perform rapid ankle dorsiflexion movements in response to an acoustic imperative stimulus. They visually fixated a projected cross and rested their arms on their thighs. Each trial started with an auditory warning stimulus (92 dB, 50 ms, 500 Hz), followed by the imperative stimulus, which was either a moderate acoustic stimulus (MAS; 82 dB, 50 ms, 1000 Hz) or a LAS (120 dB, 50 ms, 1000 Hz). Participants were instructed to perform bilateral ankle dorsiflexion movements as fast as possible following the imperative stimulus (Figure 1B). To familiarize participants with LAS, five trials consisting of warning stimuli followed by LAS were applied at the beginning. Afterwards, a training block containing five warning stimuli followed by MAS was conducted to allow the participants to practice the StartReact task. The main experiment comprised three assessment blocks, each containing 10 LAS and 20 MAS trials, with five‐minute rest periods between blocks. The interstimulus interval between warning and imperative stimuli (1.5–3 s), as well as the presentation of LAS and MAS, was pseudo‐randomized to minimize predictability of stimulus timing and type.

The lower limb movement protocol consisted of three different tasks designed to simulate daily motor activity. In the first task, participants performed a postural perturbation task while standing on a treadmill and visually fixing a cross displayed in front of them. Perturbations were induced by sudden belt accelerations (10 m/s^2^) in anterior or posterior direction, each lasting for 300 ms and reaching a target speed of 1 m/s (Figure 1C). Anterior and posterior perturbations were pseudo‐randomized to prevent anticipatory postural strategies prior to belt acceleration. For analysis, we focused on anterior belt perturbations, which resulted in a backward translation of the body, eliciting postural responses in TA muscles.

In the second task, participants performed a balance control task within the GRAIL virtual reality environment (Motek Medical B.V., CL Houten, Netherlands). A marker‐based motion capture system (Vicon system, Oxford, UK) was integrated with the GRAIL system to provide real‐time feedback on their center of mass. They adjusted their visualized center of mass to follow a visual target that oscillated vertically at 0.25 Hz, eliciting anterior (forward) and posterior (backward) body sway. The limits of postural sway were individually set to 90% of maximal anterior and posterior sway (Figure 1D).

In the third task, participants performed bilateral ankle movements while seated in a recliner. They performed visually guided dorsi‐ and plantarflexion movements while receiving real‐time feedback on ankle kinematics through the motion capture system on ankle kinematics. Movements were guided by projected visual targets (Figure 1E), with individual limits set to 90% of the participant's maximal dorsi‐ and plantarflexion, calibrated prior to each session. Ankle range of motion was assessed using three reflective markers placed on the head of the fibula (knee), lateral malleolus (ankle), and head of the 5th metatarsal (toe). Both feet followed the visual targets synchronously at a frequency of 0.25 Hz. Each task was performed in two assessment blocks. For the bilateral ankle movement and balance control task, each block consisted of 35 movement cycles over 140 s. For the postural perturbation task, each block consisted of six perturbations applied at pseudo‐randomized intervals ranging from 12 to 32 s (Table 1).

### 2.3 EMG Recordings

EMG data were collected using a wireless EMG system (Myon Aktos, Cometa Systems, Bareggio, Italy) with bipolar AG‐AgCl surface electrodes (H124SG, Kendall) placed over the SCM, DL, BB, the proximal tibialis anterior (TAp) and distal tibialis anterior (TAd). EMG electrodes for assessing TAp, TAd, DL, and BB were positioned according to SENIAM guidelines (Hermens et al. 1999). The distance between TAp and TAd electrodes was set to 10 cm to minimize crosstalk between sensors (Hansen et al. 2005). EMG electrodes for assessing the SCM were applied via muscle palpation. Placement of the electrodes was always conducted by the same experienced investigator (Nicole Sarah Holliger) to ensure accurate and reproducible electrophysiological recording. The investigator had been trained in electrode placement beforehand. EMG signals were sampled at 2000 Hz and bandpass filtered between 2 and 1000 Hz. EMG activity of TAp and TAd was recorded during the StartReact and lower limb movement tasks.

### 2.4 Data Processing and Analysis

#### 2.4.1 Startle Response Latency and StartReact Reaction Time

Startle onset latencies in the SCM, DL, and BB were determined using a custom‐written Matlab script. Muscle onset was defined as the time point at which the rectified EMG signal exceeded the mean baseline value by more than one standard deviation for at least 2 ms. Baseline EMG activity was calculated over a 100‐ms window preceding the imperative stimulus. All automatically detected onsets were visually inspected and manually corrected if necessary. Startle onset latency was defined as the interval between the onset of LAS and the identified muscle onset. Incidence of the startle response was calculated as the number of identified startles relative to the total number of LAS trials (in %).

For the StartReact and postural perturbation tasks, muscle onset latencies of TAp and TAd were determined using the same procedure described above. The only modification was that the onset was defined as the time point at which the EMG signal exceeded the mean baseline activity by two standard deviations for at least 5 ms. Reaction time in the StartReact task was defined as the interval between the onset of the imperative stimulus (LAS or MAS) and the identified muscle onset. Reaction times in the postural perturbation task were calculated between the onset of the mechanical perturbation and the identified muscle onset. No reaction times were calculated for the bilateral ankle movement and balance control tasks.

#### 2.4.2 Coherence Analysis

For coherence analysis, EMG data were full‐wave rectified. No additional band‐pass filter was applied. Data were analyzed using the Neuropsec routines Type 1 analysis (Halliday et al. 1995) (Neurospec 2.0, available at https://github.com/dmhalliday/NeuroSpec), performing discrete Fourier transformations to decompose the signals in the frequency domain. In this study, intermuscular coherence describes the comparison of the left and right side of each muscle group.

To analyze the startle reflex, intermuscular coherence was calculated between homologous SCM, DL, and BB muscles. The discrete Fourier transformations were computed using 256 samples (128 ms), with zero padding applied to achieve a segment/epoch length of 512 samples (256 ms) and a frequency resolution of 3.91 Hz. Zero padding was applied due to the limited time window of the startle reflex, thereby improving the visual resolution. However, the underlying frequency resolution is related to the original duration of the sample (i.e., 7.81 Hz). In addition to the onset detection algorithm described above, only EMG segments displaying sustained muscle activity throughout the entire duration (i.e., 128 ms for startle reflex, 256 ms for StartReact and lower limb tasks) were included in the analysis. A custom Matlab script ensured that the EMG signal exceeded the mean baseline activity by two standard deviations. EMG signals not fulfilling this criterion were excluded.

For the StartReact and lower limb movement tasks, intermuscular coherence was calculated between the left and right TAp. Further, for the StartReact task, intramuscular coherence was analyzed between TAp and TAd of the dominant leg. The dominant leg was determined by asking the participants which leg they would use to stabilize when being pushed from behind. Twelve participants showed a right dominant leg. Discrete Fourier transformations were computed using 512 samples (256 ms), with zero padding applied to yield a segment length of 1024 samples (512 ms) and a visual frequency resolution of 1.95 Hz. Underlying frequency resolution was 3.91 Hz. Although muscle activity was typically longer than 512 samples, the window for analysis was aligned to EMG onset to capture the most pronounced, initial RS drive. All lower limb movement tasks were analyzed using the same approach, as the initial EMG responses were of primary interest.

For the StartReact task, an additional analysis was performed to calculate intermuscular coherence in a moving time window of 1024 samples (512 ms), aligned to different time points relative to EMG onset. The analysis window was shifted in 10 ms increments, starting at EMG onset (0 ms) up to 80 ms after muscle onset.

Coherence analysis assesses the linear correlation between two EMG signals in the frequency domain on a scale from 0 (no linear relationship between signals x and y) to 1 (perfect linear relationship between signals x and y). The coherence function at frequency λ is defined as the absolute square of the cross spectrum ($f_{\mathrm{xy}}\left(\lambda \right)$) between the two EMG signals x and y, normalized by the product of the two autospectra of each EMG signal ($f_{\mathrm{xx}}$, $f_{\mathrm{yy}}$) (Halliday et al. 1995).

$${\left|R_{\mathrm{xy}}\left(\lambda \right)\right|}^{2}=\frac{{\left|f_{\mathrm{xy}}\left(\lambda \right)\right|}^{2}}{f_{\mathrm{xx}}\left(\lambda \right)f_{\mathrm{yy}}\left(\lambda \right)}$$

Cumulant density function characterizes the correlation between the two EMG signals in the time domain. The cumulant density function is defined as the inverse Fourier transform of the cross spectrum, and it is a function of time lag u.

$$q_{\mathrm{xy}}\left(u\right)=\int_{\;-\pi }^{\pi }f_{\mathrm{xy}}\;\left(\lambda \right)\;e^{\mathrm{i\lambda u}}\mathrm{d\lambda}$$

A central peak around zero lag indicates the synchronous activity of a sample of motor units. Cumulant density plots can be used to detect crosstalk between two signals. Crosstalk is identified as a narrow central peak and a significant high‐amplitude broadband coherence (Hansen et al. 2001; Halliday et al. 2003).

For all analyses, pooled coherence measures were calculated. It was assumed that data sets to be pooled were independent (Halliday et al. 1995). No indications for crosstalk between the EMG signals were found in our data.

Mean coherence values were calculated for the high alpha and beta bands of each task and every participant. Due to the short segment length of our coherence analysis a high frequency resolution was used (7.8 Hz for startle reflex and 3.9 Hz for StartReact and lower limb movement tasks). Therefore, only high frequencies of the alpha band were included. The high alpha band was defined from 11.7 to 15.6 Hz for the startle response and from 9.8 to 15.6 Hz for the StartReact and lower limb tasks. The beta band's range was defined from 19.5 to 31.3 Hz for the startle reflex and from 17.6 to 31.2 Hz for the StartReact and dynamic movement tasks. The differences in frequency band definition between startle vs. StartReact/dynamic tasks result from the different segment lengths and, therefore, frequency resolutions. Mean coherence was calculated by adding the coherence values from the considered frequency bins and then dividing by the number of considered frequency bins.

### 2.5 Statistical Analysis

Statistical analysis of reaction times and frequency bands was performed in R (R version 4.2.1/RStudio 2023.06.0 for Windows). The $\chi^{2}$ difference of coherence test was analyzed in Matlab. The level of significance was set at *α* = 0.05 for all statistical tests. All tests were adjusted for multiple comparisons via post hoc Bonferroni or Dunn's correction. Normality of data was assessed using the Shapiro–Wilk test.

#### 2.5.1 Response Latency and Reaction Time

Normally distributed data were assessed using parametric tests (paired *t* test, repeated measures one‐way ANOVA). Non‐normally distributed data were assessed using nonparametric statistical tests (Wilcoxon's matched‐pairs signed‐rank test; Friedman's test).

#### 2.5.2 Coherence

Coherence was considered significant when it exceeded the 95% confidence limit (CL). CL was estimated based on the assumption of independence and under the consideration of the number of segments L. For pooled data, L denotes the combined number of segments over all participants (Amjad et al. 1997).

$$\mathrm{CL}=1-{\left(0.05\right)}^{1(\left(L-1\right)}$$

To explore differences in pooled coherence data at each frequency bin between tasks (e.g., startle reflex and voluntary muscle contraction) and stimuli (e.g., StartReact with LAS or MAS), the $\chi^{2}$ extended difference of coherence test was calculated with the following equation:

$$2\left(\sum_{i=1}^{k}L_{i}{{\hat{z}}_{i}}^{2}-\frac{{\left(\sum_{i=1}^{k}L_{i}{\hat{z}}_{i}\right)}^{2}}{\sum_{i=1}^{k}L_{i}}\right)$$

The squared sum of the Fisher transformed coherency values was divided by the sum of the number of segments. This was then subtracted from the sum of the Fisher‐transformed coherency values and multiplied by 2. This computation was performed for each frequency bin. Null hypothesis was rejected if the values from $\chi^{2}$ test statistics exceeded the 100(1‐α) % CL. CL was set at the value ${\chi^{2}}_{\left(\propto ;k-1\right)}$. If the value was below this level, the null hypothesis would not be rejected at the frequency λ (Amjad et al. 1997).

#### 2.5.3 Frequency Band Analysis (High Alpha and Beta)

For the analysis of mean values of high alpha and beta bands, variance stabilizing Fisher's transform (tanh^−1^) of the magnitude of coherency was applied (Amjad et al. 1997). Linear mixed‐effects models were fitted using the lmer function of the lme4 package in R. To assess the startle reflex, a model was fitted with task (startle response, voluntary contraction) and frequency band (high alpha, beta) as fixed effects and participants as random effect. For the StartReact tasks various models were fitted. To assess intermuscular coherence of shifted analysis windows, a model was fitted with task (LAS, MAS), frequency band (high alpha, beta) and timeshift (muscle onset (i.e., 0 ms), 10, 20, 30, 40, 50, 60, 70, and 80 ms) and participants as random effect. To assess intramuscular coherence, a model with task (LAS, MAS) and frequency band (high alpha, beta) as fixed effects and participants as random effect was fitted. To investigate the differences between inter‐ and intramuscular coherence a model was fitted with task (intermuscular coherence LAS, intramuscular coherence LAS) and frequency band (high alpha, beta) as fixed effects and participants as random effect. For the comparison of StartReact and postural perturbation the following model was fitted: task (LAS, postural perturbation) and frequency band (high alpha, beta) as fixed effects and participants as random effect. For the balance and dynamic ankle movement task a model was fitted with task (balance control, ankle movement) and frequency band (high alpha, beta) as fixed effects and participants as random effect. On all models a one‐way ANOVA was performed.

## 3 Results

### 3.1 Startle Reflex

#### 3.1.1 Response Latency and Habituation

Startle onset latency was 59.1 ± 4.7 ms (median ± interquartile range (IQR)) in the SCM, 71.1 ± 10.9 ms in the DL, and 75.9 ± 8.4 ms in the BB. Startle incidence was significantly greater in the SCM (mean ± SD, 33.8% ± 28.2%), than in the DL (mean ± SD, 6.8 ± 12.0%) and BB (mean ± SD, 5.6 ± 10.1%) (Friedman's test, Friedman chi^2^ (2) = 16.27, *p* < 0.001, *W* = 0.814; post hoc Bonferroni correction: SCM vs. DL (*p* = 0.002, *r* = 1.10); SCM vs. BB (*p* < 0.001, *r* = 1.19)). Startle responses showed a significant habituation over the four assessment blocks in all three muscles: SCM (Block 1: 56.4%, Block 2: 40.0%, Block 3: 26.0%, Block 4: 13.0% (Friedman's test, Friedman chi^2^ (3) = 19.18, *p* < 0.001, *W* = 0.639)), DL (Block 1: 18.2%, Block 2: 6.0%, Block 3: 2.0%, Block 4: 1.0% Friedman's test, Friedman chi^2^ (3) = 18.68, *p* < 0.001, *W* = 0.623) and BB (Block 1: 14.5%, Block 2: 4.0%, Block 3: 2.0%, Block 4: 2.0% Friedman's test, Friedman chi^2^ (3) = 10.67, *p* = 0.014, *W* = 0.356).

#### 3.1.2 Intermuscular Coherence

**FIGURE 2:**
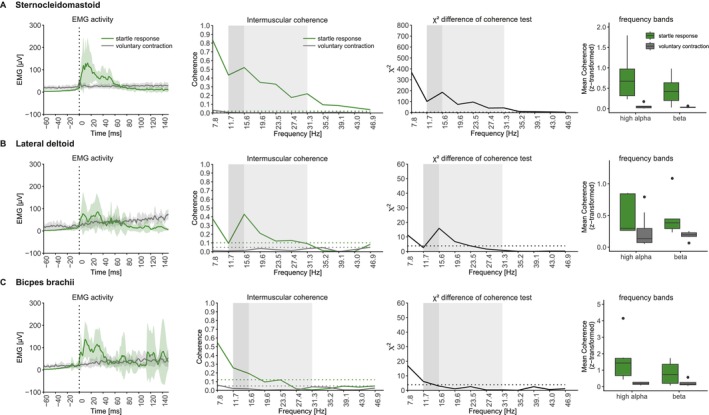
Outer left panels: Average EMG activity of startle response (green line) and voluntary contraction (grey line) in the (A) SCM, (B) DL, and (C) BB. Shaded green and grey areas indicate standard deviations. Middle left panels: Intermuscular coherence of startle response (green line) and voluntary contraction (grey line). Dotted line represents 95% confidence limit. Middle right panels: $\chi^{2}$ doc test with 95% confidence limit (dotted line). Dark grey shaded areas indicate high alpha range. Light grey shaded areas indicate beta band. Right panels: Mean coherence of high alpha and beta band of the startle response (green) and voluntary contraction (grey). doc: difference of coherence.

Mean EMG activity of the startle response and voluntary contraction across the three target muscles is shown in Figure 2. Pooled coherence analysis revealed significant differences in coherence magnitudes between the startle response and the voluntary contraction. Across all muscles, there was increased coherence at 7.8 Hz, likely reflecting the overall EMG burst envelope. Startle responses revealed enhanced intermuscular coherence magnitudes that were mainly pronounced in the high alpha and low beta band. The SCM revealed enhanced coherence estimates in the startle vs. voluntary contraction across the high alpha and beta band (*F*(1, 28) = 42.783, *p* < 0.001), with local peaks in the 10–20 Hz band, a frequency band previously related to RS motor drive (Grosse and Brown 2003) (Figure 2A). No effect of frequency range (high alpha vs. beta) was detected (*F*(1, 28) = 3.700, *p* = 0.065). In the DL, the startle response showed enhanced coherence magnitudes at 15.6 and 19.6 Hz (Figure 2B). Mean coherence analysis revealed a main effect of task (startle response vs. voluntary contraction; *F*(1, 27) = 9.872, *p* = 0.004). No effect of frequency range was detected (high alpha vs. beta; *F*(1, 27) = 0.201, *p* = 0.658). For BB, the $\chi^{2}$ difference of coherence test showed a significant difference in the high alpha band (11.7 Hz). In addition, a main effect of task was found for BB (*F*(1,26.143) = 19.153, *p* < 0.001). No effect of frequency range was evident (*F*(1,22.195) = 1.932, *p* = 0.178) (Figure 2C).

### 3.2 StartReact

#### 3.2.1 Reaction Times

Reaction time during bilateral ankle dorsiflexion was significantly faster in response to LAS (median ± IQR, 105.0 ± 12.9 ms) compared to MAS (median ± IQR, 174.8 ± 37.1 ms) (Wilcoxon's signed‐rank test, *Z* = −3.517, *p* < 0.001, *r* = 0.56). This finding confirms a substantial StartReact effect, a phenomenon thought to be mediated by RS drive.

#### 3.2.2 Intermuscular Coherence

**FIGURE 3:**
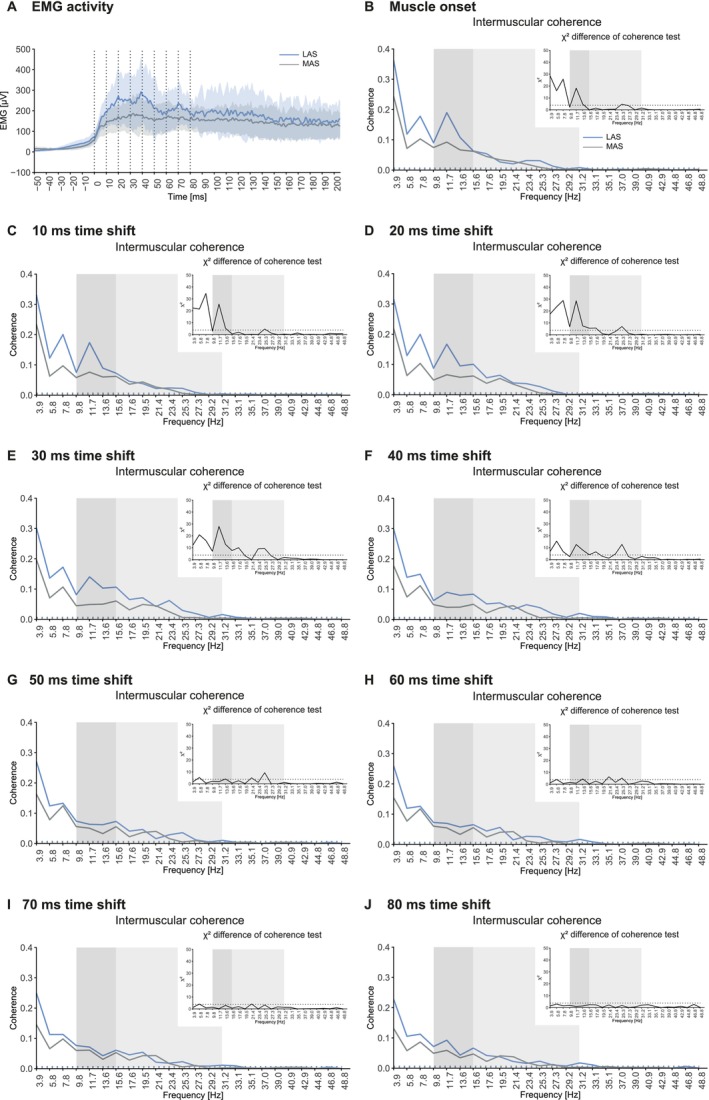
Average EMG activity of TA during StartReact and pooled intermuscular coherence estimates with corresponding $\chi^{2}$ doc test. (A) EMG activity of TA after LAS (blue line) and MAS (grey line). Shaded blue and grey areas indicate standard deviations of mean EMG activity. EMG traces of LAS and MAS were aligned to muscle onset (0 ms), rather than stimulus application, in order to allow for coherence analyses at comparable phases of muscle activation. Vertical dotted lines indicate the various time points of pooled coherence analyses. The following panels show pooled intermuscular coherence and $\chi^{2}$ doc test: (B) at muscle onset, (C) 10 ms after muscle onset, (D) 20 ms after muscle onset, (E) 30 ms after muscle onset, (F) 40 ms after muscle onset, (G) 50 ms after muscle onset, (H) 60 ms after muscle onset, (I) 70 ms after muscle onset, and (J) 80 ms after muscle onset. The 95% confidence limit is indicated by horizontal dotted line. Dark grey shaded areas indicate high alpha band. Light grey shaded areas indicate beta band. doc: difference of coherence.

Average EMG activity of the TA during the StartReact task showed broadly similar response patterns to LAS and MAS when responses were temporally aligned to their respective EMG onsets (Figure 3A). However, LAS responses exhibited enhanced amplitudes during the initial 50 ms of muscle activation, after which they converged towards MAS responses (Figure 3A). To examine intermuscular coherence across different phases of activation, the analysis window was shifted in 10 ms increments from muscle onset (0 ms) to 80 ms post‐onset (Figure 3B–J).

In the high alpha band, $\chi^{2}$ difference of coherence tests revealed an increased intermuscular coherence for LAS vs. MAS responses at 11.7 and 13.6 Hz during the initial phase of muscle activation (0–10 ms post‐onset; Figure 3B,C). Between 20 and 40 ms post‐onset, coherence was enhanced primarily in the high alpha, but also in the low beta band, with significant differences at 11.7, 13.6, 15.6, and 17.6 Hz (Figure 3D–F). Starting at 50 ms post‐muscle onset, the enhanced intermuscular coherence of LAS responses markedly attenuated, with only a single frequency bin (13.6 Hz) showing elevated coherence at 50 and 60 ms (Figure 3G,H). No significant differences were detected at 70 or 80 ms post‐onset in the high alpha band (Figure 3I,J). Analogous to the high alpha band, LAS trials also showed enhanced intermuscular coherence compared to MAS trials at low frequencies (3.9–9.8 Hz) during the first 50 ms of muscle activation, with a pronounced local peak at 7.8 Hz (Figure 3B–G). In the beta band, differences between LAS and MAS were observed during the initial 0–10 ms at 25.3 Hz. Between 20 and 70 ms post‐onset coherence at 17.6, 21.4, 23.5, and 25.3 Hz was increased in LAS responses. Thus, while the earliest phases of muscle activation revealed pronounced differences in the high alpha band, later phases were characterized by differences in the beta band around 20–25 Hz. Analysis of mean coherence during StartReact revealed a main effect of task (LAS vs. MAS; *F*(1,549) = 117.267, *p* < 0.001), and frequency band (high alpha vs. beta; *F*(1,549) = 222.075, *p* < 0.001). In addition, mean coherence of the tasks differed between the high alpha and beta range as indicated by a significant interaction effect (*F*(1,549) = 21.679, *p* < 0.001). No effect of time shift (0–80 ms) was observed (*F*(8, 549) = 1.447, *p* = 0.174; Figure S1).

#### 3.2.3 Intramuscular Coherence

**FIGURE 4:**
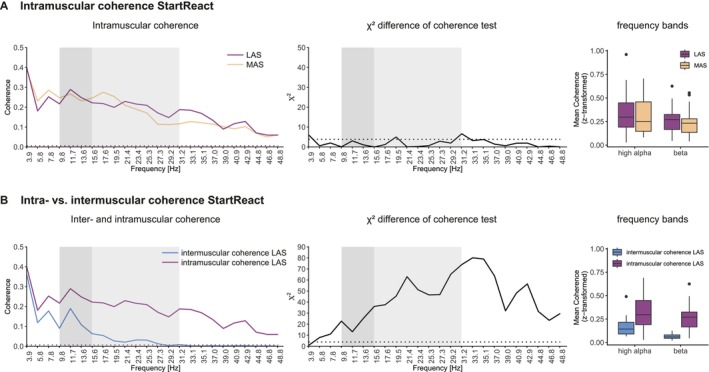
Intra‐ and intermuscular coherence of TA during StartReact. (A) Left panel: Comparison of intramuscular coherence in LAS (purple line) and MAS trials (orange line). Middle panel: $\chi^{2}$ doc. Right panel: Mean coherence of high alpha and beta band for intramuscular coherence during StartReact (LAS: purple, MAS: orange). (B) Left panel: Comparison of intra‐ and intermuscular coherence of LAS trials. Middle panel: $\chi^{2}$ doc test. Right panel: Mean coherence of high alpha and beta band for intermuscular coherence (blue) and intramuscular coherence (purple) during LAS of StartReact. Dotted line indicates 95% confidence limit. Dark grey shaded areas indicate the high alpha range. Light grey shaded areas indicate beta band. doc: difference of coherence.

Intramuscular coherence of the dominant leg was assessed between TAp and TAd. Compared to intermuscular coherence (3.2.2), intramuscular coherence showed considerably fewer differences between LAS and MAS trials (Figure 4A). Within the high alpha band, the $\chi^{2}$ difference of coherence test revealed no differences between MAS and LAS. In the beta band, differences were observed at 19.5 and 31.2 Hz (Figure 4A). Mean coherence analysis indicated no effect for task (LAS vs. MAS; *F*(1, 46) = 1.323, *p* = 0.256). However, a main effect of frequency band was detected (*F*(1, 46) = 6.094, *p* = 0.017). For the comparison between inter‐ and intramuscular coherence, TAp of the dominant leg was included for assessing intramuscular coherence as well as for assessing intermuscular coherence between the left and right TAp. When comparing inter‐ and intramuscular coherence in LAS responses, intramuscular coherence was significantly larger across all frequencies from 5.8 to 48.8 Hz, including the high alpha and beta band. This was confirmed by a significant main effect of task (intermuscular LAS vs. intramuscular LAS; *F*(1, 46) = 37.319, *p* < 0.001). In addition, a significant main effect was evident when comparing frequency bands (*F*(1, 46) = 8.623, *p* = 0.005) (Figure 4B).

### 3.3 Dynamic Movement Tasks of Lower Limbs

#### 3.3.1 Postural Perturbation Task

**FIGURE 5:**
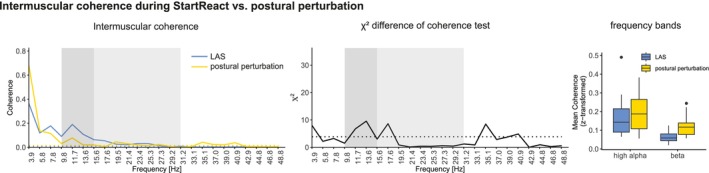
Left panel: Intermuscular coherence of TA during StartReact (blue line) and postural perturbation task (yellow line). Middle panel: $\chi^{2}$ doc test. Dotted lines indicate 95% confidence limit. Right panel: Mean coherence of high alpha and beta band during StartReact (blue) and postural perturbation (yellow). Dark grey shaded areas indicate high alpha band. Light grey shaded areas indicate beta band. doc: difference of coherence.

Perturbations applied while participants were standing on a treadmill resulted in rapid motor responses in the TA with a median reaction time of 85.4 ± 8.8 ms. Due to the putative involvement of the RS system in automatic postural response (Deliagina et al. 2014), we aimed to compare the intermuscular coherence profiles during StartReact (in LAS trials) and during postural perturbations (Figure 5). Although showing similar local peaks at 7.8 and 11.7 Hz, $\chi^{2}$ difference of coherence test revealed larger intermuscular coherence during StartReact (LAS trials) than the postural perturbation tasks at the frequency bins 11.7, 13.6, and 17.6 Hz. Further differences occurred at the frequencies 3.9, 35.1, and 40.9 Hz, where intermuscular coherence was enhanced in the postural perturbation trials compared to LAS (StartReact). Analysis of mean coherence revealed a main effect of task (LAS vs. postural perturbation; *F*(1, 46) = 4.641, *p* = 0.036) and frequency band (*F*(1, 46) = 21.731, *p* < 0.001) (Figure 5).

#### 3.3.2 Balance Control Task vs. Non‐Postural Ankle Movements

**FIGURE 6:**
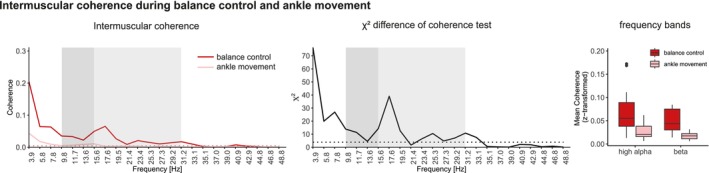
Left panel: Intermuscular coherence of TA after balance control task (red line) and ankle movement task (pink line). Middle panel: $\chi^{2}$ doc test. Dotted lines indicate 95% confidence limit. Right panel: Mean coherence of high alpha and beta band during balance control (red) and ankle movement (pink). Dark grey shaded areas indicate high alpha band. Light grey shaded areas indicate beta band. doc: difference of coherence.

Intermuscular coherence of the TA comparing a balance control tasks and non‐postural ankle movements revealed a significant difference in the high alpha band. Frequency bins from 9.8 to 15.6 Hz revealed increased coherence of balance task compared to ankle movement task. Additionally, lower frequency bins from 3.9 to 7.8 Hz and higher frequency bins from 23.4 to 31.2 Hz in the beta band showed significantly larger coherence values for the balance task compared to the ankle movement task. Mean coherence analysis confirmed a significant main effect of task (balance control vs. ankle movement), (*F*(1, 46) = 29.479, *p* < 0.001). No effect of frequency band was found (*F*(1, 46) = 0.779, *p* = 0.382) (Figure 6).

## 4 Discussion

There is increasing evidence that the RS system is a key motor system for movement control and an important substrate for motor recovery after central nervous system injury in humans. Current methods for investigating this system are limited and depend on the application of strong stimuli (e.g., StartReact paradigm, TMS). Consequently, alternative methodological approaches are needed to provide novel insights into the physiology of the RS system. In this study, we examined intermuscular coherence as potential measure of RS drive during different motor responses. Intermuscular coherence was investigated during two brainstem‐derived motor responses to identify muscle coherence patterns indicative of RS drive. Both the startle reflex and the StartReact paradigm showed enhanced intermuscular coherence in the high alpha band compared with their respective control conditions. While the initial phase of the StartReact task was characterized by a specific reproducible peak of 11.7 Hz coherence, later phases showed progressively greater coherence at higher frequencies (17.6–25.3 Hz). In addition, TA intermuscular coherence was assessed during several lower limb movements designed to reflect daily motor activities. During postural perturbation a distinct coherence peak at 11.7 Hz appeared albeit smaller than in the StartReact condition. Intermuscular coherence within both high alpha and beta band was greater during the balance control task than during a non‐postural, bilateral ankle task, suggesting RS as well as CS involvement in postural vs. non‐postural TA activity. These findings support the notion that certain aspects of RS drive may be captured by intermuscular coherence within the 10–20 Hz frequency range (Grosse and Brown 2003).

### 4.1 Intermuscular Coherence During the Startle Reflex and StartReact Paradigm

Acoustic startle reflexes were elicited in the SCM, DL and BB, with the SCM showing the highest incidence. Startle incidences and response latencies in the SCM, DL, and BB were consistent with previous reports (Brown et al. 1991; Valls‐Solé et al. 2008). As expected, startle responses showed a characteristic habituation over repeated trials (Koch 1999; Brown et al. 1991; Grosse and Brown 2003). The primary neural circuitry underlying the acoustic startle reflex is well characterized: giant RS neurons in the caudal pons get activated by neurons in the cochlear nucleus and convey excitatory drive to spinal motoneurons (Koch 1999; Yeomans and Frankland 1996; Lee et al. 1996).

We observed a marked StartReact effect in the TA during bilateral ankle dorsiflexions, confirming previous studies that demonstrated LAS‐induced reaction time shortening in the TA (Sutter et al. 2016; Hayman et al. 2025; Eilfort et al. 2025). Converging evidence from both non‐human primates (Tapia et al. 2022) and humans (Valls‐Solé et al. 1999; Neumann et al. 2025; Carlsen et al. 2004b) indicates that the StartReact effect is primarily mediated by the RS system.

Here, we used these two brainstem‐mediated motor responses to identify and quantify intermuscular coherence profiles characteristic of RS drive. To accurately capture the initial RS drive, EMG–EMG coherence was calculated within short time windows. However, this leads to methodological limitations, as short time windows reduce the frequency resolution of coherence estimates. In particular, frequencies below 10 Hz exhibit few oscillatory cycles within the short analysis window, warranting cautious interpretation of coherence values in this range. This is especially true for the 128 ms time window used for the coherence analysis of the startle response. Hence, increased intermuscular coherence in the low alpha band during startle and StartReact responses may be attributed to the short analysis windows and the overall EMG burst envelope (Figure 2). Importantly, the high alpha and beta band systematically revealed a distinct coherence peak at 11.7 Hz clearly distinguishable from the increased coherence at 7.8 Hz, suggesting that these peaks may reflect physiological sources.

The acoustic startle reflex exhibited enhanced intermuscular coherence within the high alpha band compared with voluntary contractions across all investigated muscles. A prominent coherence peak was evident at 15.6 Hz during startles in the SCM and DL, whereas the BB revealed enhanced coherence estimates at 11.7 Hz. This pattern agrees with previous findings showing enhanced intermuscular coherence between 12 and 16 Hz during startle (Grosse and Brown 2003). Coherence profiles may be partially influenced by EMG peaks of the startle response. The most prominent peak in the startle response across muscles appeared at around 10 ms. Approximating this peak with a sine wave would result in an expected coherence peak in the gamma range (around 50 Hz), a pattern not seen in any muscle. This suggests that the startle coherence profiles are not primarily shaped by individual peaks of the startle response.

During StartReact, TA intermuscular coherence was enhanced within the high alpha band in LAS compared to MAS trials. This was particularly evident during the initial phase of LAS‐related muscle activation, with the coherence peak diminishing after 40 ms. Earlier studies have demonstrated that the principal differences in EMG activity patterns during the StartReact paradigm occur within the initial 50 ms of muscle activation (Škarabot et al. 2022; Walker et al. 2024). Previous findings suggest that the initial burst of motor activity in response to LAS is predominantly mediated by the RS system (Neumann et al. 2025; Tapia et al. 2022; Škarabot et al. 2022). Thus, enhanced coherence within the high alpha band likely reflects initial RS drive elicited by LAS. The present finding of enhanced alpha band intermuscular coherence during both the startle reflex and the StartReact provides supporting evidence that enhanced intermuscular coherence within this frequency band is a marker of RS drive.

Beyond the high alpha band, additional differences in intermuscular coherence between LAS and MAS trials were observed during StartReact. LAS trials displayed a distinct coherence peak at 7.8 Hz. Although coherence at frequencies below 7.8 Hz should be interpreted with caution, previous reports have associated coherence in this range with involvement of spinal circuitries (Aguiar et al. 2018; Norton and Gorassini 2006). Alternatively, the similar time‐frequency characteristics of the 7.8 Hz and high alpha band peaks, both mainly appearing within the first 40 ms post‐muscle onset, suggest that the 7.8 Hz peak may also reflect RS drive (Chen et al. 2018). Additional coherence peaks were evident in LAS trials in the beta band (21–25 Hz). These coherence peaks might likewise be attributed to RS drive. The RS system is a complex, network‐like structure consisting of various motor nuclei (Wang 2009). While the startle response is assumed to be generated by RS neurons in the caudal pons (Yeomans and Frankland 1996; Valls‐Solé et al. 2008), the specific reticular structures mediating the StartReact effect and other motor responses are unknown. Hence, RS drive across different motor tasks may be reflected by distinct peaks across the coherence frequency spectrum. Alternatively, enhanced coherence between 17.6 and 25.3 Hz might indicate descending motor drive from other supraspinal motor centers. Previous studies have shown that increased EMG–EMG coherence in the beta band reflects CS motor control (Clark et al. 2013; Jensen et al. 2018; Zipser‐Mohammadzada et al. 2022). The pronounced appearance of the 17.6–25.3 Hz peaks between 30 and 60 ms after muscle onset suggests that these coherence peaks might originate from cortical structures that likely do not contribute to the initial muscle activation (Neumann et al. 2025). A pattern of strong initial RS drive followed by increasing CS contribution between 30 and 60 ms is consistent with a previous study reporting diminished beta band (15–30 Hz) and heightened alpha band (~8–12 Hz) coherence during movement initiation (Mehrkanoon et al. 2014).

### 4.2 Intra‐ vs. Intermuscular Coherence During the StartReact Paradigm

Intramuscular coherence of the TA in the dominant leg during StartReact showed no differences in coherence estimates within the alpha band between LAS and MAS trials. These findings contrast with the results from intermuscular coherence analysis assessing the left and right TA. Two factors may account for the discrepant findings: first, intramuscular coherence reflects synchronous oscillations among active motor units within the same muscle. In contrast, intermuscular coherence investigates synchronous oscillations between spatially separated muscle sources (left and right leg), thereby providing more specific information on bilateral supraspinal or spinal motor drive. Second, intermuscular coherence between homologous muscles is probably less sensitive to descending motor drive originating from the strongly lateralized CS tract. It can therefore be assumed that CS drive should strongly be reflected in intramuscular coherence or intermuscular coherence within the same limb (Jensen et al. 2018; Grosse et al. 2003; Grosse et al. 2002; Brown et al. 1999). In contrast, RS drive, which often descends bilaterally (Davidson and Buford 2006; Riddle et al. 2009), may be better detected by intermuscular coherence between homologous muscles.

### 4.3 Intermuscular Coherence to Characterize Neural Drive During Reactive Movements

The RS system is known to mediate rapid, explosive movements (Škarabot et al. 2022; Eilfort and Filli 2025). It is assumed that RS drive also contributes to fast, reactive movement in the context of postural corrections (Deliagina et al. 2014). Studies in cats have demonstrated that RS neurons are involved in generating compensatory postural responses following unexpected perturbations (Stapley and Drew 2009; Honeycutt et al. 2009). Recent evidence suggests that RS neurons contribute to corrective postural responses also in humans (Nonnekes et al. 2013). To further explore this, we investigated the intermuscular coherence profile of the TA muscles during sudden postural perturbations. When comparing intermuscular coherence between postural perturbations and the StartReact paradigm, we observed enhanced coherence in the alpha band and low beta band during StartReact with a peak at 11.7 Hz, which was also present, albeit smaller during postural perturbation. Coherence bins in the low gamma band (35.1–40.9 Hz) were enhanced during postural corrections compared to StartReact. These results suggest that RS drive is enhanced during StartReact compared to reactive postural corrections, a finding that may be explained by the distinct sensory stimuli triggering each response: LAS result in an activation of RS neurons through the cochlear nerve. In contrast, translational perturbation primarily engages somatosensory and vestibular inputs, likely resulting in weaker overall activation of RS neurons reflected in the high alpha band. Conversely, postural perturbations induced enhanced intermuscular coherence in the low gamma band, which may indicate greater CS involvement in postural corrections. This is in line with previous studies reporting evidence for the involvement of CS drive in generating postural responses (Nonnekes et al. 2013; Jacobs and Horak 2007; Kitatani et al. 2022). The mean coherence analysis revealed slightly higher intermuscular coherence during postural perturbations compared with the StartReact condition, particularly in the beta band. This contrasts with the findings from the pooled coherence analysis and may be attributable to methodological differences in the calculation of coherence estimates. Together, these findings support the hypothesis that postural corrections rely on an extensive interaction between CS and RS drives, whereas the StartReact effect is mainly driven by RS drive.

### 4.4 Intermuscular Coherence During Balance vs. Non‐Postural Bilateral Ankle Movements

The pivotal role of the RS system in balance control has been demonstrated in seminal preclinical studies and is assumed to apply also in humans (Deliagina et al. 2008; Prentice and Drew 2001; Brownstone and Chopek 2018). Here, we compared intermuscular coherence between a postural balance task and a non‐postural bilateral ankle movement task. We observed enhanced intermuscular coherence during the postural balance control within the high alpha band, ranging from 9.8 to 15.6 Hz. This finding agrees with a previous study examining a similar balance control task that also reported increased intermuscular coherence in low frequencies. These authors speculated that these results reflected RS involvement in balance control (Boonstra et al. 2009). In addition to the enhanced alpha band coherence, we also observed enhanced intermuscular coherence in the beta band, which may reflect additional motor drive from the motor cortex (Watanabe et al. 2018), the spinal cord (Ojha et al. 2023), or sensory afferents. Balance control presumably involves a complex interplay between various descending drives and continuous afferent inputs, enabling dynamic modulation of postural control. CS drive has been reported to be critical to complex balance control tasks (Nojima et al. 2020; Nandi et al. 2019). These different neural sources are likely reflected in the intermuscular coherence profile. Compared to the balance control task, there was decreased intermuscular coherence during non‐postural bilateral ankle movements, a pattern resembling the reduced intermuscular coherence during voluntary contractions in the SCM, DL, and BB (Figure 2). A similar pattern with reduced intermuscular coherence was reported during voluntary contractions of upper extremity muscles (Grosse and Brown 2003; Walker et al. 2019). Overall, these findings are in line with the assumption that RS drive, but also drive from other motor and sensory sources, are enhanced during balance control compared to a non‐postural task similarly involving the TA.

### 4.5 Limitations

A limitation of this study is the short segment length used for intermuscular coherence analysis. Although analyzing short segments is essential to capture the initial descending motor drive and to minimize the influence of sensory afferences on intermuscular coherence (Valls‐Solé et al. 1999; Škarabot et al. 2022), this leads to lower frequency resolution and complicates the interpretation of coherence at frequencies below 10 Hz, particularly for the startle response. However, previous work has demonstrated that short segment lengths provide sufficient signal stationarity for reliable coherence estimation (Halliday et al. 2003). Moreover, the coherence profiles observed during the startle reflex and StartReact suggest that the frequency spectrum most prone to interpretational uncertainty lies below 7.8 Hz. An additional limitation concerns the voluntary contractions used as control condition for startle responses, which revealed a relatively low EMG activity lacking the characteristic EMG burst typically seen during startle responses. This may have confounded the comparison of coherence profiles between these conditions. However, the increased intermuscular coherence within the high alpha band in LAS vs. MAS trials of the StartReact tasks (both trials involving ballistic movements) indicates that these coherence patterns do not primarily result from differential mechanical activation of the muscle across conditions. Furthermore, the rather low number of participants in this study limits the interpretability of the findings.

## 5 Conclusion

In both the startle reflex and StartReact paradigm, we observed enhanced intermuscular coherence within the high alpha band across different muscles. In the StartReact paradigm, intermuscular coherence of the high alpha band was most pronounced during the initial 40 ms of LAS‐induced muscle activation, which is assumed to be predominantly mediated by RS drive. Intermuscular coherence was also enhanced in the high alpha band during a balance control task. Increased intermuscular coherence in the beta band was observed during later phases of the StartReact paradigm and during postural corrections and balance control. Coherence at these frequencies is known to reflect CS drive, supporting the notion that the CS and RS systems act in parallel during dynamic movements. Our findings provide converging evidence that intermuscular coherence within the high alpha band reflects RS motor control during dynamic bilateral movements. Intermuscular coherence between homologous muscles seems to be particularly sensitive to capturing the diffuse, bilateral RS drive. Intermuscular coherence may be a promising noninvasive biomarker for monitoring RS drive in neurological conditions such as stroke or spinal cord injury.

## Author Contributions

**Nicole Sarah Holliger:** conceptualization, data curation, formal analysis, investigation, methodology, software, validation, visualization, writing – original draft, writing – review and editing. **Freschta Zipser‐Mohammadzada:** formal analysis, writing – review and editing. **Daniel Fabio Carpanese:** data curation, formal analysis, investigation, writing – review and editing. **Martin Schubert:** formal analysis, writing – review and editing. **Linard Filli:** conceptualization, formal analysis, funding acquisition, investigation, methodology, project administration, resources, supervision, validation, writing – review and editing.

## Funding

The study was supported by the Swiss National Science Foundation (32003B_208 110) and the Balgrist Foundation (2021‐079).

## Conflicts of Interest

The authors declare no conflicts of interest.

## Supporting information


**Figure S1:** Mean coherence of high alpha and beta band of LAS (blue) and MAS (grey) during StartReact. Analysis window was shifted starting in 10 ms increments starting from muscle onset up to 80 ms after muscle onset.

## Data Availability

All data of this study are available under the following link: https://doi.org/10.3929/ethz‐c‐000797151.
