# Supplementary figures and images for: Evidence for Increased High Alpha Intermuscular Coherence as a Measure of Reticulospinal Motor Drive
Source: Eur J Neurosci. 2026 Apr 23;63:e70517. doi: 10.1111/ejn.70517 (PMC13107094; doi:10.1111/ejn.70517)

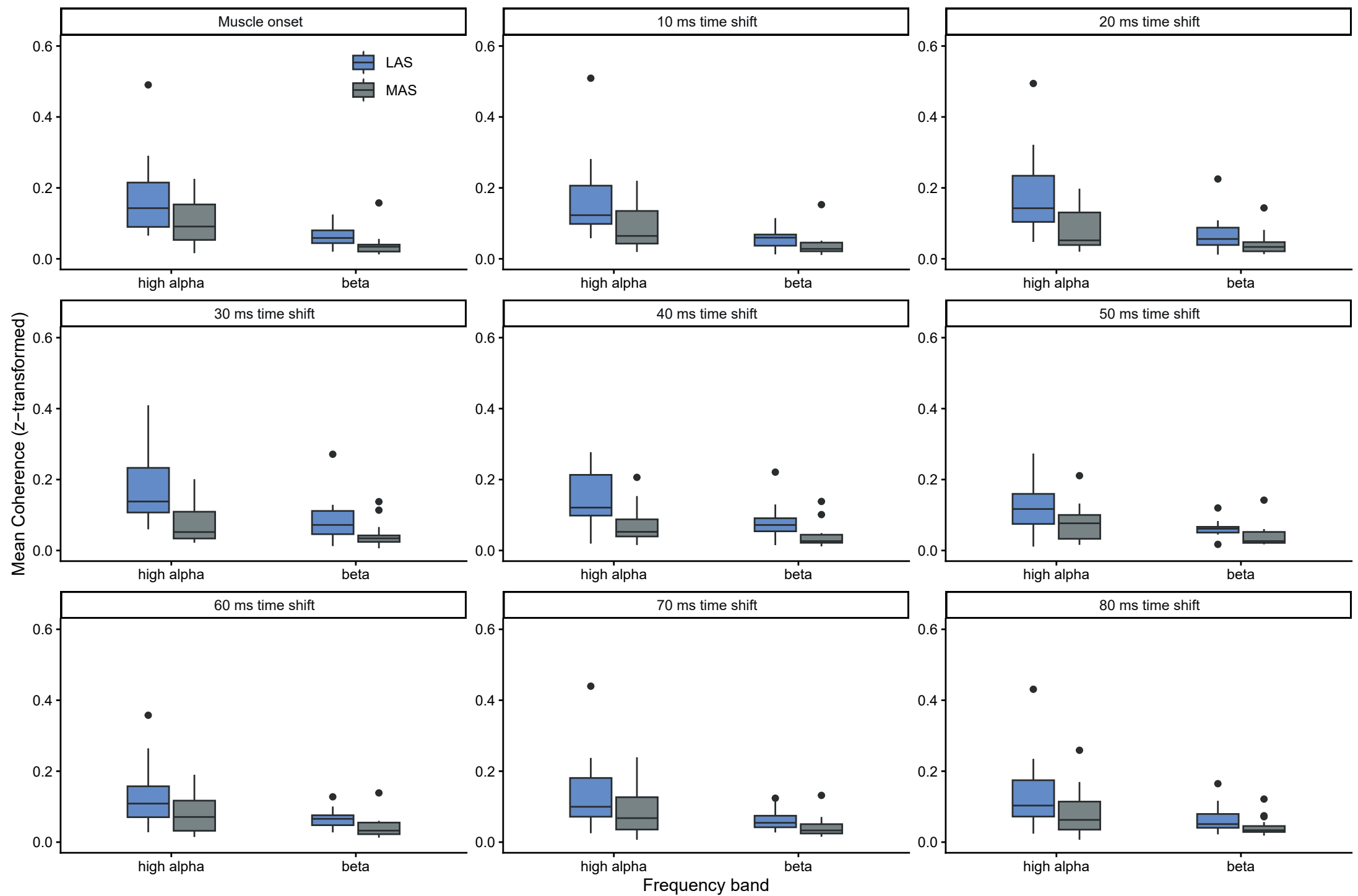

Supplement: Supplementary file 1 — Figure S1: Mean coherence of high alpha and beta band of LAS (blue) and MAS (grey) during StartReact. Analysis window was shifted starting in 10 ms increments starting from muscle onset up to 80 ms after muscle onset. [file EJN-63-0-s001.pdf]
